# Evaluation of Safety of Stewart’s Wood Fern (*Dryopteris stewartii*) and Its Anti-Hyperglycemic Potential in Alloxan-Induced Diabetic Mice

**DOI:** 10.3390/ijms232012432

**Published:** 2022-10-17

**Authors:** Uzma Hanif, Chand Raza, Iram Liaqat, Maryam Rani, Sherif M. Afifi, Tuba Esatbeyoglu, Saraj Bahadur, Sara Shahid

**Affiliations:** 1 Department of Botany, Government College University, Lahore 54000, Pakistan; mona.rani9444@gmail.com (M.R.); sarashahidrubbani@gmail.com (S.S.); 2 Department of Zoology, Government College University, Lahore 54000, Pakistan; chandraza@gcu.edu.pk (C.R.); iramliaq@hotmail.com (I.L.); 3 Pharmacognosy Department, Faculty of Pharmacy, University of Sadat City, Sadat City 32897, Egypt; sherif.afifi@fop.usc.edu.eg; 4 Institute of Food Science and Human Nutrition, Gottfried Wilhelm Leibniz University of Hannover, Am Kleinen Felde 30, 30167 Hannover, Germany; 5 College of Forestry, Hainan University, Haikou 570228, China; sirajbahadur14@gmail.com

**Keywords:** anti-diabetic, diabetes mellitus, ethnomedicine, in vivo, metabolic disorder, toxicity

## Abstract

Diabetes has become a critical challenge to the global health concerns. Cytotoxicity and development of resistance against available drugs for management of diabetes have shifted the focus of global scientific researchers from synthetic to herbal medications. Therefore, the current study was conducted to investigate the possible anti-hyperglycemic potential of *Dryopteris stewartii* using Swiss albino mice. To evaluate any possible toxic effect of the plant, acute oral toxicity test was performed while the anti-diabetic effects of aqueous and ethanol extracts at 500 mg/kg, positive, negative and normal control were assessed simultaneously. The anti-diabetic study revealed that aqueous extract has higher anti-diabetic potential than ethanol extract while lowered blood glucose level at second week reaching 150 mg/dL, exerting stronger anti-diabetic effects, compared to ethanol extract (190 mg/dL). Oral glucose tolerance findings revealed that aqueous extract decreased blood glucose level by −0.41-fold, compared to ethanol extract showing a decrease by only −0.29-folds. The histopathological evaluation of liver and pancreas of all groups revealed normal cell architecture with no morphological abnormalities. These results suggested the possible use of *D. stewartii* as anti-diabetic herbal drug in near future. However, these recommendations are conditioned by deep mechanistic studies.

## 1. Introduction

Diabetes mellitus (DM) is a group of metabolic disorders characterized by inability to properly utilize macromolecules i.e., carbohydrates, proteins and lipids that ultimately lead to hyperglycemic conditions with increasing possibilities of complication in vascular system. It is a heterogeneous class of diseases associated with metabolism resulting from faulty insulin secretion and/or action, leading to eventually pave the way toward hyperglycemia [1]. DM is probably a type of endemic disease that can be inherited or developed later due to mal-production of insulin by the pancreas, associated with insulin resistance at peripheral receptors or inefficiency of insulin to perform its role [2]. In such condition, a diabetic body cannot normally regulate blood glucose level, aside from insulin deficiency, resulting in aggravated abnormal production of fats, proteins and carbohydrates [3]. According to recent reports, around 150 million people on earth are diabetic with a probability of increment to reach 300 million before the year 2025. About 8.5% of adult people were diagnosed with diabetes in 2014, and around 1.6 million mortalities were linked to diabetes in 2017 [4]. Being among the top 10 countries where diabetes is most prevalent, Pakistan is ranked 7th in the world with the highest diabetic pervasiveness. The recent occurrence of diabetic people in Pakistan was recorded at 7.6–11%, and is expected to reach 15% by the year 2030, shifting Pakistan to 4th position globally.

In prolonged cases of hyperglycemia disorders like neuropathy, nephropathy, retinopathy and cardiovascular problems have been reported [4]. Hyperglycemia and hyperlipidemia are two main constituents or causative factors of DM. Prolonged hyperglycemia is implicated with a disturbing lipid profile by changing serum enzymes that ultimately modify the total body mass index [5,6]. There are two types of hyperglycemia; Type-1 and type-2 diabetes, triggered by a decreased level of insulin and inefficient utilization of insulin, respectively [7]. About 95% of the total diabetic patients are type-2, causing hyperglycemic complications in about 246 million persons [8,9,10]. Oxidative stress caused by hyperglycemia induces free radicals playing an important role in the development and progression of DM [11,12,13,14,15,16]. Catalase and dismutase are some antioxidant enzymes used in averting Alloxan toxicity along with some non-enzymatic hydroxyl radical scavengers [17]. Alloxan (2,4,5,6-pyrimidinetetrone) is a by-product of pyrimidine oxygenation. Alloxan-induced hyperglycemia in rodents mimics the DM features, and hence, is widely used in preclinical studies. Alloxan causes devastating effects on the pancreas by damaging the *β*-cells involved in insulin production [18]. Islet *β*-cells show indifference towards glucose (i.e., presence or absence), even at a high concentration, when Alloxan causes an abrupt increase in insulin secretion.

The World Health Organization (WHO) had great restrictions on the use of ethnomedicines for control and treatment of DM [19,20]. Around 1200 or more plants have been exploited as traditional medicine [21,22], owing to their hypoglycemic effects with about 800 plants were scientifically reported as potential evidence-based medicine to manage DM [23]. Originally derived from *Galega officinalis*, metformin is a well-known hypoglycemic drug with a historical background, providing a vivid example of studying medicinal plants for the development of pharmaceuticals [24,25,26,27,28].

The wood fern genus *Dryopteris* (ca. 225 species) [29] is one of the largest genera in Dryopteridaceae, rich in untapped members and deserves further phytochemical study [30]. A detailed review of the literature also supports the folk use of *Dryopteris* plants [31]. It is helpful for pain, epilepsy, rheumatism, cure of snake bite and diabetic treatment. The Genus *Dryopteris* have a total of over 250 identified compounds, including phenolic glycosides, flavonoids phloroglucinol, steroids, terpenoids and phenyl propanoid. *Dryopteris stewartii* is used in the treatment of many diseases like respiratory disorders (i.e., asthma, bronchitis, emphysema, pneumonia), intestinal ulcers, stomach, urinary ailments and skin disorders by the methods of decoction and infusion [32,33]. *D. stewartii* is commonly found in forests at mid to high elevations. *D.* *stewartii* is distributed in the Himalayas, Afghanistan, Pakistan, India and Nepal. The plant body is erect and branching Rhizome [31].

The current study investigates the effect of *D. stewartii* extracts on blood glucose levels of Alloxan-induced diabetic mice, acute toxicity of *D. stewartii* extracts and histological evaluation of vital organs.

## 2. Results

### 2.1. Acute Oral Toxicity Evolution

To evaluate the safety of plant extracts, 500 mg/kg of both ethanol and aqueous extracts were administered orally. All groups were observed for 14 days following the dose administration. Neither death nor any toxic signs were witnessed. The mortality rate was zero, which showed that 500 mg/kg was safe to use. Thus, 500 mg/kg doses of both extracts were selected for later experimentation.

#### 2.1.1. Effect of Aqueous and Ethanol Extracts on Body Weight

As depicted in Table 1, the mean body weight ± SD of three groups including normal control and experimental groups (received ethanol and aqueous extracts) was measured. There was a non-significant (F_2,6_ = 0.584; *p* = 0.587) difference among the mean body weights of three groups.

#### 2.1.2. Effect of Plant Extracts on Organ Weight

**Figure 1:**
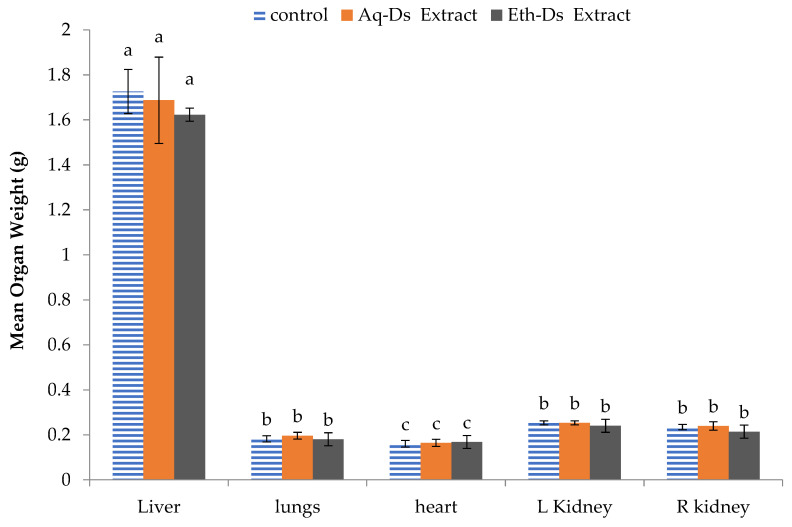
Mean organ weight of normal and extract-treated mice. No statistical difference was observed among the mice from control, Aq-DS or Eth-DS extract-treated mice liver, lungs, heart and kidneys. Groups: Eth-DS: Ethanolic extract of DS, Aq-DS: Aqueous extract of DS. Letter a, b or c above each bar is the standard error which indicates that the values are significant.

There was a non-significant difference among the mean weights of the individual organs of normal control and extract treated groups including liver (F_2,6_ = 2.860; *p* = 0.134), lungs (F_2,6_ = 3.545; *p* = 0.096), heart (F_2,6_ = 0.362; *p* = 0.710), left kidney (F_2,6_ = 4.750; *p* = 0.058), and right kidney (F_2,6_ = 1.699; *p* = 0.260). The graphical representation along with the standard deviation is shown in Figure 1.

#### 2.1.3. Histolopathological Assessments

A histological examination of five organs, including liver, kidney, heart, lung and pancreas, was performed to investigate any disease manifestation or cellular damage.

**Figure 2:**
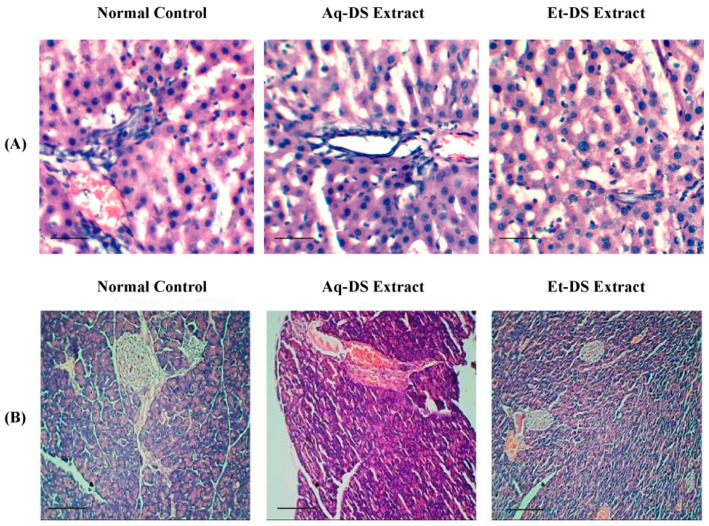
Histopathological photomicrographs of normal control, Aq-DS (aqueous extract of DS group), and Et-DS (ethanolic extract of DS group) extracts administered at 500 mg/kg single doses in the liver and pancreas. (**A**) Liver: normal hepatic cells and well-preserved cell organelles without pathological alterations are evident in hematoxylin and eosin-stained sections (5 µm thick) at 40× magnification. (**B**) Pancreas: normal islets of Langerhans without pathological alterations are evident in hematoxylin and eosin-stained sections at 10×. Scale Bars: (**A**,**B**) = 0.2 cm.

The histological examination of the liver tissues of normal control and extract treated groups revealed that there was no pathological abnormality in liver. Photomicrographs of the liver from each group (Figure 2A) showed normal hepatocytes without any lesion. Slides expressed prominent nuclei, central vein and sinusoids. Histopathological photomicrographs of the pancreas (Figure 2B) of control and extract treated groups showed normal islet cell morphology and architecture. Normal islets of Langerhans were visible surrounded with exocrine portion. No necrotic or inflammation were observed. Islet cells were considerably large in diameter and compactly arranged with small intercellular spaces.

**Figure 3:**
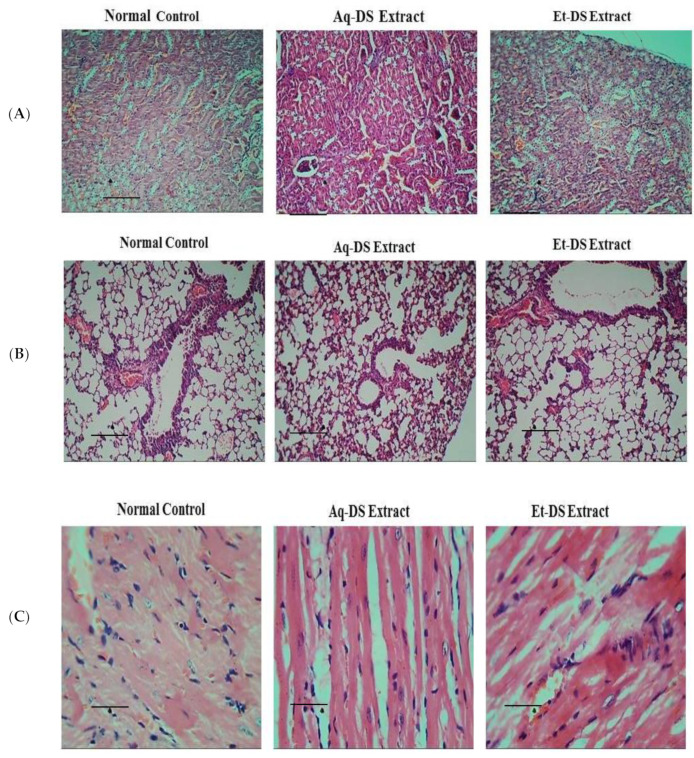
Histopathological photomicrographs of normal control, Aq-DS (aqueous extract of DS group) and Et-DS (ethanolic extract of DS group) extracts administered at 500 mg/kg, single dose in (**A**) Kidney, normal structures of renal corpuscles and renal tubules without pathological signs are visible in Hematoxylin and Eosin stained sections (5 µm thick) at 40× magnification; (**B**) Normal histology of lung showing bronchioles and alveoli without pathological signs in Hematoxylin and Eosin stained sections (5 µm thick) at 40× magnification; (**C**) Clear cardiac muscle fibers without pathological signs are evident in Hematoxylin and Eosin stained sections (5 µm thick) at 40×. Scale Bars: (**A**,**B**) = 1.5 cm, (**C**) = 0.8 cm.

Photomicrographs of kidney sections (Figure 3A) from normal control and *D. sterwartii* extracts treated groups showed normal renal histo-architecture. Normal structure of renal corpuscles with renal tubule and glomeruli was evident in kidney sections from all three groups. No shrinkage or fragmentation of glomeruli was observed. Photomicrographs of the lungs of control and extract treated groups (Figure 3B) showed normal histology. No abnormalities in bronchioles and alveoli were observed in lung sections from each group. Normal epithelium and blood vessels were also evident, showing that both aqueous and ethanol extracts of *D. sterwartii* have no toxic effects on lung tissues of experimental animals. Photomicrographs of the heart tissues of control and extract treated groups showed normal histology. Normal architecture of cardiac myofibers (Figure 3C) was observed. There was no visible degeneration or fragmentation of myofibrils, demonstrating that both aqueous and ethanol extracts had no toxic effects on the heart tissues of experimental animals.

### 2.2. Anti-Hyperglycemic Activity

#### 2.2.1. Effects of Extracts and Standard Drug on Body Weight

**Figure 4:**
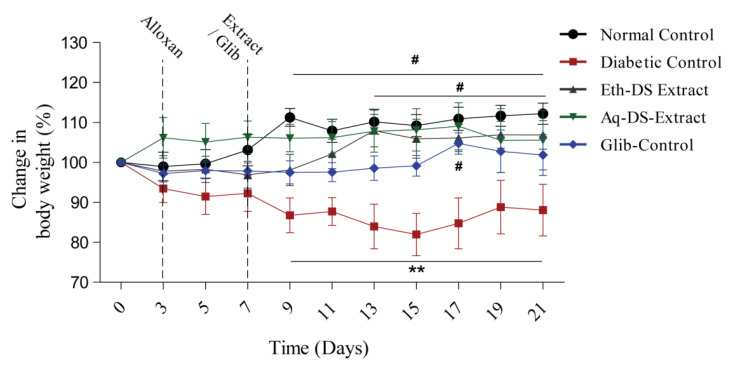
Variability in the body weight percentage (Mean ± SD, n = 6) of diabetic mice treated with glibenclamide and DS extracts, # *p* < 0.05, ** *p* < 0.01. (Eth-DS. Ethanolic extract of DS, Aq-DS. Aqueous extract of DS, Glib. glibenclamide treated group).

Figure 4 demonstrated that there was a significant difference (*p* < 0.01) in the body weight percentage among normal control and aqueous extract treated group during 9–21 days, compared to diabetic control. Likewise, a prominent difference (*p* < 0.01) was observed in the body weight percentage among the ethanol extract treated group during 13–21 days, compared to diabetic control. Furthermore, the glibenclamide treated group revealed a significant difference (*p* < 0.05), compared to diabetic control during 9–21 days.

#### 2.2.2. Effects of Extracts and Standard Drug on Blood Glucose Levels

**Figure 5:**
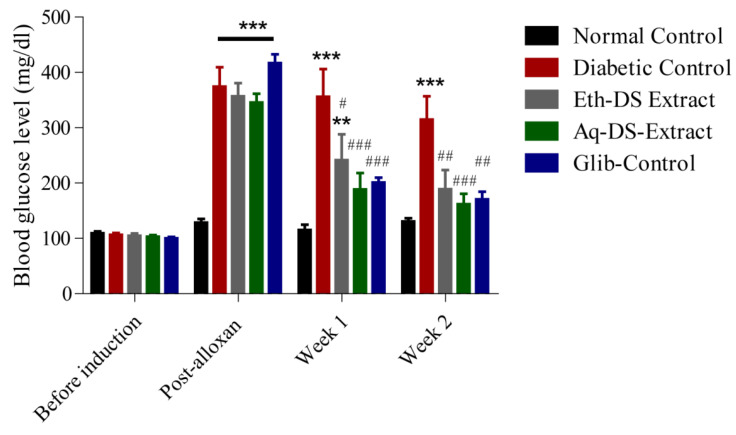
Effects of DS extracts (ethanol and aqueous) and glibenclamide on blood glucose levels of alloxan induced diabetic mice, expressed as mean ± SD (n = 6 per group), ** *p* < 0.01, *** *p* < 0.001 compared to normal control, # *p* < 0.05, ## *p* < 0.01, ### *p* < 0.001 compared to diabetic control. (Eth-DS: Ethanolic extract of DS, Aq-DS: Aqueous extract ofDS, Glib: glibenclamide treated group).

Before the induction of diabetes, results revealed normal blood glucose levels (95–110 mg/dL) in all groups (Figure 5). However, upon induction of diabetes, significantly higher blood glucose levels (*p* < 0.001) were observed in all experimental groups (Diabetic Control, Eth-DS Extract, Aq-DS Extract and Glib-Control) except for normal control. After one week of treatment, a significant decrease in blood glucose levels (*p* < 0.001) of three experimental groups i.e., Eth-DS extract, Aq-DS extract and Glib-Control was detected, compared to diabetic control. Similar results in the blood glucose levels were observed in the second week of treatment. However, the group treated with Aq-DS extract potentially had a lowered blood glucose level at the second week reaching 150 mg/dL and exerted stronger anti-diabetic effects, compared to Eth-DS extract (190 mg/dL). Groups treated with both extracts showed comparable blood glucose levels with the glibenclamide treated mice during the experiment.

#### 2.2.3. Oral Glucose Tolerance Test

**Figure 6:**
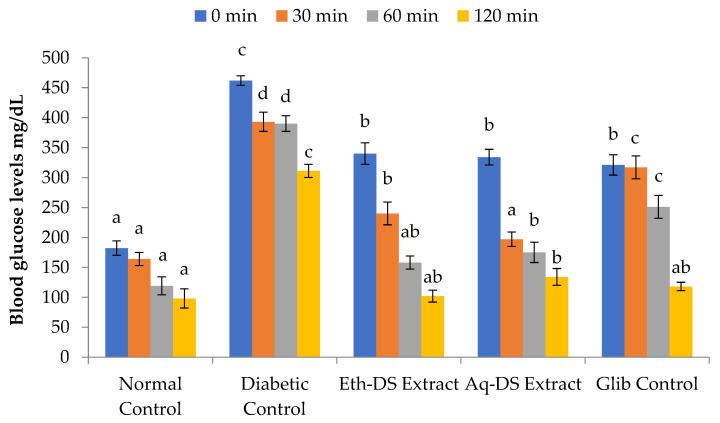
Comparative study of variability in glucose tolerance levels at various time intervals. A relatively maintained level of blood glucose in Eth-DS, Aq-DS extracts treated groups and in Glib control mice in sharp contrast to elevated blood glucose levels in diabetic mice. Different letters indicate significant difference among groups. (Eth-DS: Ethanolic extract of *D. stewartii*, Aq-DS: Aqueous extract of *D. stewartii*, Glib: glibenclamide treated group).

Higher blood glucose levels were recorded for all experimental groups and diabetic control, compared to normal control (Figure 6) at 0 min (*p* < 0.001). Similarly, a gradual significant decrease in the blood glucose levels of all groups compared to diabetic control was observed from 30 to 120 min. After 30 min of glucose administration, the aqueous extract treated group revealed a decrease in blood glucose level by −0.41-fold, compared to ethanol extract and glibenclamide treated groups showing a decrease by only −0.29 and −0.03-fold, respectively. However, blood glucose levels reached normal in all experimental groups except the diabetic control after 120 min. The difference among the blood glucose levels of all four groups, except the diabetic control, after 120 min was non-significant (*p* = 0.438).

#### 2.2.4. Histology of Liver and Pancreas

**Figure 7:**
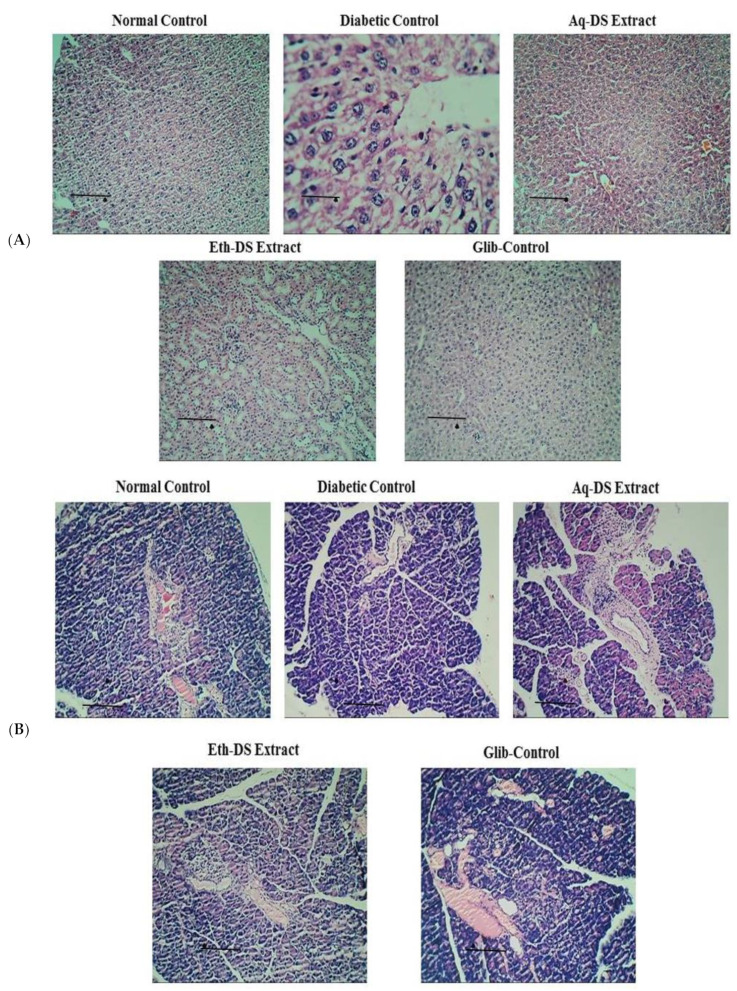
Photomicrographs of the effect of different treatments on (**A**) Liver architecture. Liver of control group showed normal hepatocytes architecture. Photomicrograph of diabetic group exhibited cell necrosis and sinusoidal dilation. Photomicrograph of aqueous and ethanol extract (500 mg/kg) treated groups revealed almost same morphology as normal group with normal hepatocytes architecture (H & E stain, ×10 & 40). (**B**) Pancreas, Pancreas of normal control mice showed normal islets surrounded with exocrine portion. Pancreas sections of diabetic mice revealed necrosis and shrinkage of islet cells. In photomicrographs from aqueous extract (500 mg/kg) treated group, enlarged islets were evident. Improved islet size and normal pancreatic structure was observed in pancreas of ethanol extract (500 mg/kg) and glibenclamide (5 mg/kg) treated groups (H & E stain, 10× & 40×). Scale Bars: (**A**,**B**) = 0.5 cm. (Eth-DS: Ethanolic extract of DS, Aq-DS: Aqueous extract of DS, Glib: glibenclamide treated group).

A histological assessment of the liver tissues from normal control, diabetic control, positive control and extract treated groups was done to observe pathological abnormalities (Figure 7A). Photomicrographs of the liver from normal control group showed normal hepatocytes with prominent nuclei. Normal arrangements of hepatic cells were observed without any lesion. Photomicrographs of liver sections of diabetic mice showed abnormal structure. In diabetic mice, the liver cells were more vacuolated. Diabetic liver cells showed degeneration of parenchyma cells with dilated sinusoids and central vein. Daily administration of aqueous and ethanol extracts at 500 mg/kg resulted in less vacuolization. Photomicrographs of liver from aqueous extract treated group showed normal appearance of hepatic cells with mild dilation of sinusoids. In contrast, hepatic cells of ethanol extract treated group showed a nearly normal appearance with less sinusoidal congestion and slight necrosis. Photomicrograph from positive control group treated with glibenclamide (5 mg/kg) exhibited normal hepatic cell arrangement. The liver histology of aqueous and ethanol extract treated groups was comparable with glibenclamide treated group.

A histological examination of the pancreatic tissues from normal control, diabetic control, positive control and extract treated groups was performed to observe pathological abnormalities (Figure 7B). Normal structure and architecture of islet cells were evident in photomicrographs of normal control group. Normal population of alpha and beta cells in islets of Langerhans was also observed. However, diabetic control group showed damaged islets with reduced size. There was an increase in islet size in aqueous extract treated diabetic group with the presence of normal acini. Ethanol extract (500 mg/kg) treated diabetic group and glibenclamide (5 mg/kg) treated diabetic mice showed improved islet cell architecture and morphology with negligible necrosis.

## 3. Discussion

The number of diabetic patients has been increasing at an alarming rate, globally Poor nutritional intake, lethargic lifestyle and unhygienic environmental conditions have been scrutinized as the major causes. Moreover, complications of diabetes have also worsened due to unawareness of the disease management, insufficient health facilities and misuse of the orthodox/conventional medications The present study was conducted to evaluate the anti-hyperglycemic potentials of aqueous and ethanol extracts of *D. stewartii* on diabetic experimental animal (Swiss albino mice). Acute oral toxicity was assessed to check the safety of both extracts. Neither death nor any toxic symptoms were witnessed in extract treated mice for 14 days following single oral dose administration. These results indicated the safe nature of *D. stewartii*, encouraging the continuation of the hypoglycemic experiment. In agreement with the present acute toxicity results, other researchers [33,34] also reported similar findings in *Artemisia afra* based anti-diabetic studies. Moreover, during the conduction of acute oral toxicity test, body and organ weights of extract treated groups remained normal, indicating the harmless nature of *D. stewartii*. In compliance with the current experiment findings, other articles [35,36] also reported similar results in *Stephania japonica* and *Astraeushygo metricus*. However, one article documented contrasting results of *Lantana camara* and *Dryopteris ramose* based toxicity studies [37,38,39,40,41]. Furthermore, the histological assessment of five organs (liver, lung, kidney, pancreas and heart) of normal control and extract treated groups were keenly examined through photomicrographs, revealing normal cell architecture. Also, no characteristic deformities were observed in the experimental groups. These findings suggest the safe nature of *D. stewartii.* In accordance with the present results, other researchers [38,39] also reported similar findings in *Rhoicissus tridentate* and *Momordica charantia* L.

After the induction of diabetes through Alloxan, the anti-hyperglycemic effect of plant extracts was also assessed. Significantly higher blood glucose levels in all experimental groups were observed after intraperitoneal administration of alloxan monohydrate, revealing the induction of diabetes mellitus. In this regard, blood glucose levels of extracts and standard drug treated groups were compared with normal and diabetic control. There was a considerable decrease in the blood glucose levels in all experimental groups (Aq-DS extract, Eth-DS extract and Glib-Control) owing to anti-hyperglycemic potential of standard drug and plant extracts. In accordance with the current results, others [6] also reported the significant decrease in blood glucose levels of diabetic mice after treatment with the extracts of *Pterisvitata* L. and *Adiantum philippense* Linn. These results supported the possible anti-hyperglycemic potential of *D. stewartii*. The anti-diabetic potential of the plant is due to its bioactive constituents. However, aqueous extract revealed potentially higher anti-diabetic results compared to ethanol extract. The possible reason behind this phenomenon could be due to the presence of more polar anti-hyperglycemic phytochemicals in aqueous based extract. To investigate the possible reasons behind this finding, deeper and mechanistic studies are inevitable. To evaluate the general glucose metabolism in case of abrupt intake of glucose, an oral glucose tolerance test was conducted. After oral administration of glucose, the rise in blood glucose levels was gradually decreased in all experimental groups except diabetic control, indicating the malfunctioning of the pancreas [40]. Similar findings were also reported [6] in *Albizia odoratissima* Benth; however, *Dryopteris ramosa* also showed the high phenolic contents (199.2 mg gallic acid/g extract) with repression of Cholinesterase activity belonging to the same genus [41] has confirmed the use of this plant as therapeutic agent is also in accordance with our results. Different plants showed various medicinal benefits in different ways, either as crude extracts or fractions exploited for the treatment of many diseases [42,43,44,45,46]. Thus, the proper authentication of the desired plant [44,45,46] will provide authentic scientific knowledge [47,48,49] for its better exploitation in different disorders [50,51].

Last but not the least, a histopathological evaluation was carried out to assess the possible effects of both extracts on the morphology of the liver and pancreas. Photomicrographs revealed improved hepatocytes and pancreatic cells needed for proper functioning of respective organs. These results encourage the possible use of *D. stewartii* (GC.Herb.Bot.1900) as a potential anti-diabetic agent in near future.

## 4. Materials and Methods

### 4.1. Collection of Plant Material and Authentication

The fresh and disease-free plant, *D. stewartii,* was provided by the Herbarium of Department of Botany, GC University Lahore, Pakistan. Plants were transferred safely to the General Botany Laboratory, Government college University Lahore, Pakistan and identified by the taxonomic experts based on standard morphological characters [30], and a voucher specimen number (GC.Herb.Bot.1900) was submitted in Dr. Sultan Ahmad Herbarium, Government College University, Lahore Pakistan.

### 4.2. Preparation of Extracts

After collection, the plant material was carefully washed with running water to eliminate impurities and shade dried for one week. The drying procedure was done away from sun exposure and with appropriate air flow. The completely dried plant material was then ground into powder using an electronic grinder. The dried material was sealed in an airtight container and stored at room temperature until use.

Preparation of Aqueous Extract of *D. stewartii*: Aqueous extract was prepared with distilled water (10% *w*/*v*) and prepared by adding 30 g powder in 300 mL of distilled water. The solution was placed in a flask orbital shaker at 100 rpm speed and 25 °C for 24 h. Then, the solution was filtered by Whatman filter paper No. 1. The filtrate was collected in a beaker. The resultant aqueous extract was concentrated under reduced pressure to obtain *D. stewartii* crude aqueous extract. Extract was stored at 4 °C until used for experiment.

Preparation of Ethanol Extract of *D. stewartii*: The ethanol plant extract was prepared with 99.9% ethanol solution (10% *w*/*v*) and prepared by adding 30 g powder in 300 mL of ethanol. The solution was placed in a flask orbital shaker at 100 rpm speed and 25 °C for 24 h. After 24 h of maceration, the solution was filtered by Whatman filter paper No.1. The filtrate was collected in a beaker. The resultant ethanol extract was concentrated under reduced pressure to obtain *D. stewartii* crude ethanol extract. The extract was stored at 4 °C until used for experiment.

### 4.3. Acute Oral Toxicity Evaluation

#### 4.3.1. Animals

Before the commencement of the experiment, ethical conditions on the use of laboratory animals were considered and followed as approved by the Institutional Bioethics Committee of Government College University Lahore (AEC/GCU/1082). Adult male Swiss albino mice (6–7 weeks old) were obtained from the Animal Housing Facility of University of Veterinary and Animal Sciences Lahore, Pakistan. The animals were acclimatized to laboratory conditions for one week. Animals were kept in standard cages with metallic grill tops and provided with clean husk bedding. They were accommodated under standard temperature (25 ± 2 °C) and relative humidity with 12 h light/dark cycle, with unlimited supplies of water and rodent diet.

#### 4.3.2. Animal Grouping and Experimental Design

To measure the safety of both plant extracts, the acute oral toxicity evaluation of the extracts was determined before experimentation. For the acute oral toxicity evaluation, fifteen male Swiss albino mice were divided into three groups (n = 5) in the following manner: Group1-Normal control (NC):This group did not receive any extract but was provided with a standard diet and water. It received distilled water (10 mL/kg) and acted as control group. Group 2-Ethanol extract of *D. stewartii* (Eth-DS): This group received 500 mg/kg of ethanol extract and allowed free access to feed and water. The extract was suspended in distilled water and administered orally. Group 3-Aqueous extract of *D. stewartii* (Aq-DS): Group 3 received 500 mg/kg of aqueous extract and allowed free access to feed and water. The extract was dissolved in distilled water and administered orally. After a single oral administration of plant extracts, the mice were observed for signs of possible toxicity and mortality for 14 days. The animals were weighed on daily basis during experiment. The animals were also visually observed to analyze the changes in the fur, skin and eyes.

#### 4.3.3. Anesthesia Preparation

Mice anesthesia was induced using 100 mg/kg Ketamine and 10 mg/kg Xylazine cocktail. The mice were intraperitoneally injected with a 10 mL/kg cocktail and became anesthetized within 2 min. The tail of each anesthetized mouse was pinched for confirmation of anesthesia.

#### 4.3.4. Terminal Anesthesia

The cervical dislocation of anesthetized mice was performed to euthanize the animals for organ procurement.

#### 4.3.5. Dissection

To procure the different organs (heart, liver, kidney, pancreas and lungs), dissection was conducted. The organs were weighed individually for further study. Autoclaved surgical tools were used under strict hygienic conditions.

#### 4.3.6. Histopathology

The organs were preserved in 10% neutral buffered formalin (NBF) for further histological assessment. The fixed organs were dehydrated in serial grades of ethanol, cleared in xylene and embedded in paraffin wax. The tissues were sectioned (5 mm thick) and were mounted on slides. Hematoxylin and Eosin staining was performed, as reported [22]. previously. The stained sections were microscopically evaluated and photographed with a camera (Panasonic DMC-FH3) under light microscope (Olympus CX23; Tokyo, Japan).

### 4.4. Anti-Hyperglycemic Effect

#### 4.4.1. Animals

For the evaluation of the anti-hyperglycemic activity, 42 Swiss albino mice (6–7 weeks old) (25–30 g) were selected. Prior to the experiment, the mice were allowed to acclimatize to laboratory conditions for one week under standard conditions.

#### 4.4.2. Animal Grouping and Experimental Design

To proceed in the experiment, diabetic mice were selected and divided into 5 groups consisting of eight to ten mice per each group. The groups were designed and maintained as follows: Group 1-Normal control (NC): Ten healthy Swiss albino mice were kept in this group and fed a normal diet and water. Group 2-Diabetic control (DC): This group consisted of eight diabetic mice and fed a normal diet and water only. Group 3-Ethanol extract treated group of *D. stewartii* (Eth-DS): Eight diabetic mice were placed in ethanol group. The mice received daily ethanol extract 500 mg/kg suspended in distilled water for 14 days. The prepared ethanol extract dose 10 mL/kg was given through oral route to each mouse on a daily basis. They were also given a standard pellet diet and tap water. Group4-Aqueous extract treated group of *D. stewartii* (Aq-DS): This group comprised of eight diabetic mice. These mice received daily 500 mg/kg aqueous extract dissolved in distilled water for 14 days. The prepared aqueous extract dose 10 mL/kg was given through oral route to each mouse on a daily basis. The animals had free access to feed and water. Group 5-Glibenclamide treated group (Glib): Eight diabetic mice were selected and received 5 mg/kg glibenclamide on a daily basis. The prepared dose 10 mL/kg was given through intraperitoneal injections. This group served as a positive control group. The mice were fed a standard pellet diet and tap water [36].

#### 4.4.3. Induction of Hyperglycemia

In this experiment, alloxan monohydrate was used as a diabetogenic agent. Alloxan (2,4,5,6-tetraoxypyrimidine; 2,4,5,6-pyrimidinetetrone) is an oxygenated derivative of pyrimidine. Alloxan selectively destroys *β*-cells of pancreas with irreversible necrosis generating chronic hyperglycemia.

Before the induction of hyperglycemia, the mice were fasted overnight (had access to water). A dose of 200 mg/kg body weight freshly prepared alloxan monohydrate dissolved in 10 mL/kg body weight distilled water was injected intraperitoneally [35]. Immediately after administration of Alloxan, a standard diet was provided to animals, preventing alloxan-induced hypoglycemia due to release of insulin from pancreatic *β*-cells. On the 5th day after induction, the animals were fasted for 6 h and the blood glucose level was monitored to confirm the induction of diabetes. The glucose level was measured using Certeza GL-110 digital glucometer. Blood was taken by making a small incision at end of the tail vein. Mice with 300 mg/dL blood glucose level were considered diabetic and selected for assessment of anti-hyperglycemic activity [35].

#### 4.4.4. Assessment of Body Weight

In all experimental groups, the body weight of each mouse was measured initially before the induction of diabetes. After the successful onset of disease, the body weight of each animal was recorded at regular intervals of 2 days throughout the experiment period.

#### 4.4.5. Determination of Fasting Blood Glucose Level

Before the induction of diabetes, blood glucose levels of all experimental groups were measured after 6 h fasting to obtain basal readings. Blood glucose levels were also monitored at 5th day after the hyperglycemia induction by alloxan administration. Thereafter, successful induction of hyperglycemia, fasting glucose levels were determined every week in two weeks period.

Blood samples were taken by making a small incision at the end of the tail vein with the help of sharp surgical scissors after sterilizing the tail with 10% alcohol. The blood drop was placed on a test strip and inserted in a calibrated Certeza GL-110 digital glucometer.

#### 4.4.6. Oral Glucose Tolerance Test

At the end of 14 days, an oral glucose tolerance test was performed to monitor the clearance of an oral glucose load from body. The test was performed according to MMPC (Mouse Metabolic Phenotyping Center) protocol [36].

Mice were fasted overnight, then 2 g glucose/kg body weight was administered by oral gavage.

Blood glucose levels were measured at 0, 30, 60 and 120 min after the administration of glucose with the help of digital glucometer.

Blood samples were taken by making a small incision at the end of the tail vein by sharp surgical scissors.

#### 4.4.7. Terminal Euthanasia

At the end of experiment, the animals were euthanized to collect tissues. Before the dissection of each animal, 200 µL anesthetic solution (xylazine/ketamine) was administered intraperitoneally. Cervical dislocation was performed to euthanize the animals for dissection. The liver and pancreas were procured from each animal.

#### 4.4.8. Histopathology

The organs were preserved in 10% neutral buffered formalin (NBF) for further histological assessment. For the histological evaluation, slides were prepared to assess the cell morphology and architecture of organs. After fixation, the tissues were dehydrated in increased concentrations of alcohol, cleared in xylene and embedded in paraffin wax. Multiple 5 mm sections from each block were mounted on slides and stained with hematoxylin and eosin. Stained sections were microscopically evaluated and photographed.

### 4.5. Statistical Analysis

Data was expressed as mean ± standard deviation (S.D) and analyzed using two-way ANOVA followed by Bonferroni Post hoc test using GraphPad Prism (v5.0, San Diego, CA, USA).

## 5. Conclusions

The findings of the current study suggested that extracts of *D. stewartii* are potentially harmless to the animal model (Swiss albino mice), as indicated by the detailed histological investigations. The unidentified bioactive constituents exerting anti-hyperglycemic effects need to be investigated using modern analytical tools. Further, these results recommended possible incorporation of *D. stewartii* extracts in anti-diabetic formulation albeit, after further biological investigation. Additionally, the exact mode of action regarding anti-hyperglycemic activity of *D. stewartii* is undiscovered, and needs to be explored. Overall, the current study is a valuable reference for further clinically relevant investigations.

## Figures and Tables

**Table 1:** Mean body weight ± SD of normal and extract treated groups.

	Groups			*p*-Value
	Normal Control	Eth-DS	Aq-DS	
Mean Body Weight (g)	27.94 ± 0.504	28.00 ± 0.416	27.29 ± 0.320	0.587

Eth-DS: Ethanolic extract of *Dryopteris stewartii*; Aq-DS: Aqueous extract of *Dryopteris stewartii.*

## Data Availability

Not Applicable.

## References

[1] ADA (American Diabetes Association) (2001). Report of the expert committee on the diagnosis and classification of diabetes mellitus. Diabetes Care.

[2] Rasool S., Faheem M., Hanif U., Bahadur S., Taj S., Liaqat F., Pereira L., Liaqat I., Shaheen S., Shuaib M. (2022). Toxicological effects of the chemical and green ZnO NPs on *Cyprinus carpio* L. observed under light and scanning electron microscopy. Microsc. Res. Technol..

[3] Ramachandran S., Asokkumar K., Maheswari M.U., Ravi T., Sivashanmugam A., Saravanan S. (2011). Investigation of antidiabetic, antihyperlipidemic, and in vivo antioxidant properties of *Sphaeranthus indicus* Linn. in type 1 diabetic rats: An identification of possible biomarkers. Evid. Based Complement. Altern. Med.

[4] World Health Statistics (2016). Monitoring Health for the SDGs Sustainable Development Goals.

[5] Tafesse T.B., Hymete A., Mekonnen Y., Tadesse M. (2017). Antidiabetic activity and phytochemical screening of extracts of the leaves of *Ajugaremota* Benth on alloxan-induced diabetic mice. BMC Complement. Altern. Med..

[6] Kumar S., Kumar V., Prakash O. (2011). Antidiabetic, hypolipidemic and histopathological analysis of *Dilleniaindica* (L.) leaves extract on alloxan induced diabetic rats. Asian Pac. J. Trop. Biomed..

[7] Marshal W., Bangret S.K. (2004). Clinical Chemistry.

[8] Mycek J.M., Harvey S., Chape P.C. (2000). Insulin and Oral Hypoglycemic Drugs in Lippincott’s Illustrated Reviews, Pharmacology.

[9] Lenzen S., Panten U. (1988). Alloxan: History and Mechanism of Action. Diabetologia.

[10] Oberley L.W. (1988). Free Radicals and Diabetes. Free Radic. Biol. Med..

[11] Jorns A., Munday R., Tiedge M., Lenzen S. (1997). Comparative toxicity of alloxan, *N*-alkyl alloxan and ninhydrin to isolated pancreatic islets in vitro. J. Endocrinol..

[12] Chaudhry S.R., Akram A., Aslam N., Asif M., Wajid M., Kinfe T., Jabeen Q., Muhammad S. (2016). Antidiabetic and antidyslipidemic effects of *Heliotropium strigosum* in rat models of Type I and Type II diabetes. Acta Pol. Pharm..

[13] Szkudelski T. (2001). The mechanism of alloxan and streptozotocin action in B cells of the rat pancreas. Physiol. Res..

[14] Salahuddin M., Jalalpure S.S. (2010). Antidiabetic activity of aqueous fruit extract of *Cucumis trigonus* Roxb. in Streptozotocin-induced diabetic rats. J. Ethnopharmacol..

[15] Johansen J.S., Harris A.K., Rychly D.J., Ergul A. (2005). Oxidative stress and the use of antioxidants in diabetes: Linking basic science to clinical practice. Cardiovasc. Diabetol..

[16] Matough F.A., Budin S.B., Hamid Z.A., Alwahaibi N., Mohamed J. (2012). The role of oxidative stress and antioxidants in diabetic complications. Sultan Qaboos Univ. Med. J..

[17] Etuk E.U. (2010). Animals models for studying diabetes mellitus. Agric. Biol. J. N. Am..

[18] Jebur A.B., Mokhamer M.H., El-Demerdash F.M. (2016). A Review on oxidative stress and role of antioxidants in diabetes mellitus. Austin Endocrinol. Diabetes Case Rep..

[19] World Health Organisation (2018). Diabetes WHO Fact Sheet.

[20] Hussain A., Ali I. (2016). Diabetes mellitus in Pakistan: A major public health concern. Arch. Pharm. Pract..

[21] Tsang M.W. (2012). The management of type 2 diabetic patients with hypoglycaemic agents. ISRN Endocrinol..

[22] Jain A.K., Mehta S.C., Shrivastava N.M. (2005). Hypoglycemic and antihyperglycemic effects of newly synthesized sulfonyloxy derivatives of azalactone in normal and Alloxan diabetic rabbits. Indian J. Pharmacol..

[23] Pandey A., Tripathi P., Pandey R., Srivatava R., Goswami S. (2011). Alternative therapies useful in the management of diabetes: A systematic review. J. Pharm. Bioallied Sci..

[24] Aggarwal N., Shishu B. (2011). A review of recent investigations on medicinal herbs possessing anti-diabetic properties. J. Nutr. Disord. Ther..

[25] Li W.L., Zheng H.C., Bukuru J., De Kimpe N. (2004). Natural medicines used in the traditional Chinese medical system for therapy of diabetes mellitus. J. Ethnopharmacol..

[26] Fabricant D.S., Farnsworth N.R. (2001). The Value of Plants Used in Traditional Medicine for Drug Discovery. Environ. Health Perspect..

[27] Paul T., Apte K.G., Parab P.B., Das B. (2017). Role of *Adiantum philippense* L. on glucose uptake in isolated pancreatic cells and inhibition of adipocyte differentiation in 3T3-L1 cell line. Pharmacogn. Mag..

[28] Pryer K.M., Schneider H., Smith A.R., Cranfill R., Wolf P.G., Hunt J.S., Sipes S.D. (2001). Horsetails and ferns are a monophyletic group and the closest living relatives to seed plants. Nature.

[29] Fraser-Jenkins C.R. (1986). A classification of the genus *Dryopteris* (Pteridophyta and Dryopteridaceae). Bull. Br. Mus. (Nat. Hist.).

[30] Smith A.R., Pryer K., Schuettpelz E., Korall P., Schneider H., Wolf P. (2006). A classification for extant ferns. Taxon.

[31] Hoshizaki B.J., Wilson K.A. (1999). The cultivated species of the fern genus *Dryopteris* in the United States. Am. Fern J..

[32] Li C.X., Lu S.G. (2006). Phylogenetics of Chinese *Dryopteris* (Dryopteridaceae) based on the chloroplast rps4-trnS sequence data. J. Plant Res..

[33] Irfan M., Jan G., Jan F.G., Murad W. (2022). Floristic diversity and chorotype analysis of the pteridophytes of Pakistan. J. Anim. Plant Sci..

[34] Han X., Li Z., Li C.Y., Jia W.N., Wang H.T., Wang C.H. (2015). Phytochemical constituents and biological activities of plants from the genus *Dryopteris*. Chem. Biodives..

[35] Zehad A., Islam G.J., Rashid M., Juthy N.J., Zannah S. (2017). Antidiabetic and antihyperlipidemic activities of methanolic leaf extract of *Stephania japonica* in Alloxan Induced Diabetic Rats. Pharm. Pharmacol..

[36] Biswas G., Acharya K. (2013). Hypoglycemic activity of ethanolic extract of *Astraeus hygrometricus* (pers.) Morg. inalloxan-induced diabetic mice. Int. J. Pharm. Pharm. Sci..

[37] Pour B.M., Latha L.Y., Sasidharan S. (2011). Cytotoxicity and oral acute toxicity studies of *Lantana camara* leaf extract. Molecules.

[38] Hanif U., Mukhtar A., Khan Z.D., Hussain T., Jabeen R., But G.Y. (2016). Anatomical study of two Hydrophytes–*Pistia stratiotes* L. and *Centella asiatica* L. Urban. Bio Logia.

[39] Xu X., Shan B., Liao C.H., Xie J.H., Wen P.W., Shid J.Y. (2015). Anti-diabetic properties of *Momordica charantia* L. polysaccharide in alloxan-induced diabetic mice. Int. J. Biol. Macromol..

[40] Paul T., Das B., Apte K.G., Banerjee S., Saxena R.C. (2012). Evaluation of anti-hyperglycemic activity of *Adiantum philippense* Linn, a pteridophyte in alloxan induced diabetic rats. J. Diabetes Metab..

[41] Alam F., Khan S.H.A., Bin Asad M.H.H. (2021). Phytochemical, antimicrobial, antioxidant and enzyme inhibitory potential of medicinal plant *Dryopteris ramosa* (Hope) C. Chr. BMC Complement. Med. Ther..

[42] Hussain M., Liaqat I., Ali N.M., Arshad N., Hanif U., Sajjad S., Sardar A.A., Awan U.F., Khan F.S. (2021). Antibacterial and bacteriostatic potential of coelomic fluid and body paste of *Pheretima posthuma* (Vaillant, 1868) (Clitellata, Megascolecidae) against ampicillin resistant clinical bacterial isolates. Braz. J. Biol..

[43] Gao Z., Ali Z., Zhao J., Qiao L., Lei H., Lu Y., Khan I.A. (2008). Phytochemical investigation of the rhizomes of *Dryopteris crassirhizoma*. Phytochem. Lett..

[44] Khan M.T., Azhar I., Shehzadi N., Hussain K., Parveen S., Hanif U. (2020). Morphological, microscopic, and physicochemical studies of *Diospyros montana*. Microsc. Res. Technol..

[45] Younis S., Shaheen S., Zaib M., Harun N., Khalid S., Hussain K., Hanif U., Khan F. (2020). Scanning electron microscopic screening of 20 medicinally important Asteroideae taxa. Microsc. Res. Technol..

[46] Yin P., Wang Y., Yang L., Sui J., Liu Y. (2018). Hypoglycemic effects in alloxan-induced diabetic rats of the phenolic extract from mongolian oak cups enriched in ellagic acid, kaempferol and their derivatives. Molecules.

[47] Lin X., Xu Y., Pan X., Xu J., Ding Y., Sun X., Song X., Ren Y., Shan P.F. (2020). Global, regional, and national burden and trend of diabetes in 195 countries and territories: An analysis from 1990 to 2025. Sci. Rep..

[48] Fox L.A., Pfeffer E., Stockman J., Shapiro S., Dully K. (2020). Medical Neglect in Children and Adolescents with Diabetes Mellitus. J. Adolesc. Trauma.

[49] Cassidy E.M., O’Halloran D.J., Barry S. (1999). Insulin as a substance of misuse in a patient with insulin dependent diabetes mellitus. BMJ.

[50] Pandhare R.B., Sangameswaran B., Mohite P.B., Khanage S.G. (2011). Antidiabetic activity of aqueous leaves extract of *Sesbania sesban* (L.) Merr. In streptozotocin induced diabetic rats. Avicenna J. Med. Biotechnol..

[51] Jaiswal Y.S., Tatke P.A., Gabhe S.Y., Vaidya A.B. (2017). Antidiabetic activity of extracts of *Anacardium occidentale* Linn. leaves on n-streptozotocin diabetic rats. J. Tradit. Complement. Med..

