# Correction: Hanif et al. Evaluation of Safety of Stewart’s Wood Fern (*Dryopteris stewartii*) and Its Anti-Hyperglycemic Potential in Alloxan-Induced Diabetic Mice. *Int. J. Mol. Sci.* 2022, *23*, 12432

**DOI:** 10.3390/ijms27052346

**Published:** 2026-03-03

**Authors:** Uzma Hanif, Chand Raza, Iram Liaqat, Maryam Rani, Sherif M. Afifi, Tuba Esatbeyoglu, Saraj Bahadur, Sara Shahid

**Affiliations:** 1 Department of Botany, Government College University, Lahore 54000, Pakistan; mona.rani9444@gmail.com (M.R.); sarashahidrubbani@gmail.com (S.S.); 2 Department of Zoology, Government College University, Lahore 54000, Pakistan; chandraza@gcu.edu.pk (C.R.); iramliaq@hotmail.com (I.L.); 3 Pharmacognosy Department, Faculty of Pharmacy, University of Sadat City, Sadat City 32897, Egypt; sherif.afifi@fop.usc.edu.eg; 4 Institute of Food Science and Human Nutrition, Gottfried Wilhelm Leibniz University of Hannover, Am Kleinen Felde 30, 30167 Hannover, Germany; 5 College of Forestry, Hainan University, Haikou 570228, China; sirajbahadur14@gmail.com

In the original publication [1], there was a mistake in Figure 2A. In Figure 2A, there is a slight duplication of the pictures. Figure 2 was accidentally uploaded and used in our manuscript. The correct Figure 2 appears below.

In the original publication, there was also another error, as the authors forgot to include the Institutional Review Board Statement in the manuscript. This has now been corrected. The correct paragraph appears below.

**Institutional Review Board Statement**

This study was conducted and approved by the Institutional Bioethics Committee of Government College University Lahore (approval code: AEC/GCU/1082; approval date: 27 November 2019).

The authors state that the scientific conclusions are unaffected. This correction was approved by the Academic Editor. The original publication has also been updated.

## Figures and Tables

**Figure 2:**
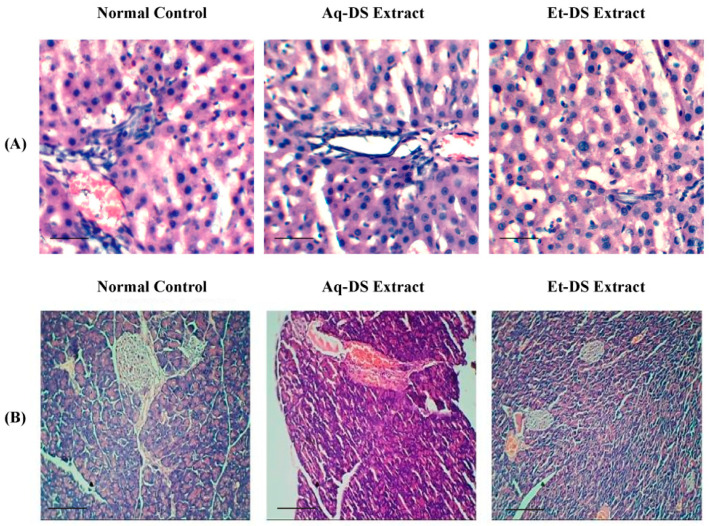
Histopathological photomicrographs of normal control, Aq-DS (aqueous extract of DS group), and Et-DS (ethanolic extract of DS group) extracts administered at 500 mg/kg single doses in the liver and pancreas. (**A**) Liver: normal hepatic cells and well-preserved cell organelles without pathological alterations are evident in hematoxylin and eosin-stained sections (5 µm thick) at 40× magnification. (**B**) Pancreas: normal islets of Langerhans without pathological alterations are evident in hematoxylin and eosin-stained sections at 10×. Scale Bars: (**A**,**B**) = 0.2 cm.
